# Supplementary material for: The Depressiveness, Quality of Life and NEO-FFI Scale in Patients with Selected Genodermatoses
Source: J Clin Med. 2024 Mar 12;13(6):1624. doi: 10.3390/jcm13061624 (PMC10971189; doi:10.3390/jcm13061624)
Supplement: Supplementary file 1 [file jcm-13-01624-s001.zip › jcm-2878417-supplementary/jcm-2878417-supplementary.pdf]

Supplementary Table S1. Detailed patient characteristics and their confirmed mutations.

ID	Age [years]	Sex	Genodermatosis type	Genotype	Scales on exposed parts of the body	Fraction of the body surface covered by lesions	The whole body is covered with scales	The dominant color of the scales	Skin itching	Redness	Exfoliation	Infiltration	Pain (VAS)	Increased sweating	Sleep disorders	Ectropion	Closing the eyelids	Feeling of ear plugging	Hearing loss	Performance status (Karnofsky scale)
1	34	M	ARCI	ALOX12B*	+	0,8	+	Beige	5	8	7	+	0	Absolutely no sweating	-	-	+	+	+	90
2	32	M	EEC	TP63*	-	0,02	-	White	2	2	2	+	0	N	-	-	-	-	-	90
3	41	M	EV like	ND	+	0,8	+	Brown	8	8	10	-	0	+	+	+	-	+	-	50
4	16	M	EI	KRT10*	+	0,98	+	White	1	6	2	+	2	-	-	-	+	+	-	100
5	59	F	EI	ND	-	1	-	Multicolored	8	7	10	-	2	Absolutely no sweating	+	-	+	+	-	100
6	47	M	IWC	KRT10*	+	0,9	+	White	9	10	9	+	8	-	+	+	-	+	+	25
7	24	F	LI	TGM1*	+	1	+	Multicolored	3	4	10	+	0	Absolutely no sweating	-	+	-	+	-	80
8	57	M	LI	ND	+	1	+	Multicolored	3	6	8	+	8	-	+	+	-	+	-	35
9	42	F	LI	ND	+	1	+	Multicolored	10	9	10	-	8	-	+	+	-	+	+	30
10	26	F	LI	TGM1*	+	1	+	Multicolored	7	4	7	-	4	+	-	+	-	-	+	85
11	59	F	LI	ND	+	1	+	Multicolored	5	1	5	-	1	-	-	+	-	+	-	40
12	35	F	LI	ND	-	0,8	-	Transparent	4	6	9	-	1	+	-	-	+	-	-	100
13	18	F	LI	TGM1*	-	0,04	-	Brown	8	10	10	+	0	-	-	+	+	-	-	90
14	43	F	LI	ND	+	1	+	Multicolored	9	6	10	+	8	+	+	-	+	+	-	95
15	34	F	LI	ND	+	0,9	-	Multicolored	4	5	8	-	1	+	-	-	-	-	-	95
16	31	M	KP	ND	-	0,04	-	Yellow	5	5	4	+	8	N	-	-	-	-	-	70
17	18	M	KP	KRT1*	-	0,06	-	Beige	4	3	3	+	5	+	-	-	-	-	-	90
18	26	M	KP	JUP*	-	0,1	-	Yellow	3	6	7	-	7	-	-	-	+	-	-	50
19	43	F	KP	KRT1*	-	0,06	-	Yellow	4	5	3	+	2	N	+	-	-	-	-	80
20	48	F	KP	KRT9*	-	0,04	-	Yellow	3	2	1	+	4	N	-	-	-	-	-	60
21	36	F	IV	ND	-	0,6	-	Beige	8	3	8	-	1	N	-	-	+	+	-	80
22	47	F	IV	ND	+	1	-	Beige	4	3	3	-	7	+	-	-	+	-	-	100

23	25	F	IV	ND	-	0,2	-	Multicolored	2	5	6	-	1	-	-	-	+	-	-	100
24	44	F	IV	ND	-	0,5	-	Brown	2	1	4	-	1	-	+	-	+	-	-	100
25	51	F	IV	FLG*	-	0,9	+	Beige	6	4	10	+	0	N	-	-	+	-	-	100
26	62	F	IV	ND	+	1	-	Multicolored	5	4	5	-	3	+	+	-	+	-	+	70
27	34	F	IV	ND	+	0,98	+	Brown	6	1	7	-	2	N	-	-	+	-	-	100
28	24	F	IV	ND	-	1	-	Brown	7	8	5	-	3	-	+	-	+	+	-	95
29	79	F	IV	ND	+	0,01	+	White	3	4	8	-	2	+	-	-	+	+	+	50
30	36	F	IV	ND	+	1	+	Beige	3	6	4	-	0	+	-	-	+	-	-	100

ND no data

ARCI Autosomal recessive congenital ichthyosis

EEC Ectrodactyly ectodermal dysplasia

EI Erythrodermic ichthyosis,

EV Epidermodysplasia verruciformis

I Ichthyosis

LI Lamellar Ichthyosis

IWC Ichthyosis with confetti

KP Non-epidermolytic palmoplantar keratoderma

IV Ichthyosis vulgaris

F Female

M Male

Supplementary Table S2. The relationship between demographic, social, educational, and clinical factors and quality of life assessed with the Dermatology Life Quality Index.

Patient characteristics	Values	Quality of life		*p*
		Severely or very severely reduced [n=13]	Normal, slightly or moderately reduced [n=17]	
**Sex**	Men<br>Women	9 (42.9%)<br>4 (44.4%)	12 (57.1%)<br>5 (55.6%)	0.7478
**Age [years]**	Median [IQR] (Min-Max)	42 [32.7-47]	34 [25.7-48.7]	0.4893
**Place of residence**	City<br>Village	8 (44.4%)<br>5 (41.7%)	10 (55.6%)<br>7 (58.3%)	0.8215
**Education**	Primary, Vocational, Secondary<br>Higher	8 (53.3%)<br>5 (33.3%)	7 (46.7%)<br>10 (66.7%)	0.4612
**Scales on exposed parts of the body**	No<br>Yes	5 (35.7%)<br>8 (50%)	9 (64.3%)<br>8 (50%)	0.6756
**% of the body surface covered by lesions**	Median [IQR] (Min-Max)	100% [62.5%-100%]	80% [5.5%-98.5%]	0.1439
**The whole body is covered in scales**	No<br>Yes	7 (43.7%)<br>6 (42.9%)	9 (56.2%)<br>8 (57.1%)	0.7489
**Color of scales**	Beige, yellow or brown	9 (40.9%)	13 (59.1%)	0.9778

	White	4 (50%)	4 (50%)	
**Skin itching**	Median [IQR] (Min-Max)	5 [3.7-8.2]	4 [2.7-6]	0.1085
**Redness**	Median [IQR] (Min-Max)	6 [5.7-8.2]	4 [2-5]	0.0019*
**Exfoliation**	Median [IQR] (Min-Max)	8 [4.7-10]	7 [3.7-8]	0.2626
**Infiltration**	No	8 (47.1%)	9 (52.9%)	0.9210
	Yes	5 (38.5%)	8 (61.5%)	
**Pain (VAS)**	Median [IQR] (Min-Max)	3 [0.7-8]	1 [0.7-2.5]	0.1159
**Increased sweating**	No	7 (35%)	13 (65%)	0.3619
	Yes	6 (60%)	4 (40%)	
**Ectropion**	No	8 (36.4%)	14 (63.6%)	0.3893
	Yes	5 (62.5%)	3 (37.5%)	
**Sleep disorders**	No	5 (25%)	15 (75%)	0.0133*
	Yes	8 (80%)	2 (20%)	
**Inability to fully close the eyelids**	No	8 (47.1%)	9 (52.9%)	0.9210
	Yes	5 (38.5%)	8 (61.5%)	
**Feeling of ear plugging**	No	7 (41.2%)	10 (58.8%)	0.9210
	Yes	6 (46.2%)	7 (53.8%)	
**Hearing loss**	No	10 (41.7%)	14 (58.3%)	0.9266
	Yes	3 (50%)	3 (50%)	
**Performance status (Karnofsky scale)**	Median [IQR] (Min-Max)	80 [46.2-96.2]	90 [77.5-100]	0.2421

* - statistically significant result

Supplementary Table S3. The relationship between personality inventory assessed using the NEO-FFI questionnaire and quality of life assessed using the Dermatology Life Quality Index.

**Personality Inventory**	**Level**	**Quality of life**		***p***
		**Severely or very severely reduced [n=13]**	**Normal, slightly or moderately reduced [n=17]**	
**Openness to experience**	Low<br>Moderate<br>High	0 (0%)<br>5 (62.5%)<br>8 (40%)	2 (100%)<br>3 (37.5%)<br>12 (60%)	0.2445
**Neuroticism**	Low<br>Moderate<br>High	0 (0%)<br>3 (27.3%)<br>10 (55.6%)	1 (100%)<br>8 (72.7%)<br>8 (44.4%)	0.2214
**Agreeableness**	Low<br>Moderate<br>High	1 (100%)<br>6 (75%)<br>6 (28.6%)	0 (0%)<br>2 (25%)<br>15 (71.4%)	0.0400*
**Extroversion**	Moderate<br>High	4 (44.4%)<br>9 (42.9%)	5 (55.6%)<br>12 (57.1%)	0.7478
**Scrupulousness**	Low<br>Moderate<br>High	6 (66.7%)<br>7 (33.3%)	3 (33.3%)<br>14 (66.7%)	0.1983

* - statistically significant result

Supplementary Table S4. The relationship between demographic, social, educational, and clinical factors with the occurrence of depression (according to Beck`s scale).

Patient characteristics	Values	Depression		*p*
		No [n=17]	Yes [n=13]	
**Sex**	Men<br>Women	11 (52.4%)<br>6 (66.7%)	10 (47.6%)<br>3 (33.3%)	0.7478
**Age [years]**	Median [IQR] (Min-Max)	34 [24.7-48]	42 [33.2-47.2]	0.3352
**Place of residence**	City<br>Village	10 (55.6%)<br>7 (58.3%)	8 (44.4%)<br>5 (41.7%)	0.8215
**Education**	Primary, Vocational, Secondary<br>Higher	8 (53.3%)<br>9 (60%)	7 (46.7%)<br>6 (40%)	1.0000
**Scales on exposed parts of the body**	No<br>Yes	8 (57.1%)<br>9 (56.2%)	6 (42.9%)<br>7 (43.7%)	0.7489
**% of the body surface covered in lesions**	Median [IQR] (Min-Max)	90% [17.5%-100%]	80% [4%-100%]	0.4391
**The whole body is covered in scales**	No<br>Yes	10 (62.5%)<br>7 (50%)	6 (37.5%)<br>7 (50%)	0.7489
**Color of scales**	Beige, yellow or brown<br>White	13 (59.1%)<br>4 (50%)	9 (40.9%)<br>4 (50%)	0.9778

**Skin itching**	Median [IQR] (Min-Max)	4 [3-5]	7 [3.7-8.2]	0.0238*
**Redness**	Median [IQR] (Min-Max)	5 [3-6]	6 [3.5-8.2]	0.3421
**Exfoliation**	Median [IQR] (Min-Max)	5 [3-8]	9 [6.2-10]	0.0518
**Infiltrations**	No<br>Yes	9 (52.9%)<br>8 (61.5%)	8 (47.1%)<br>5 (38.5%)	0.9210
**Pain (VAS)**	Median [IQR] (Min-Max)	1 [0-3.5]	2 [1-8]	0.1950
**Increased sweating**	No<br>Yes	12 (60%)<br>5 (50%)	8 (40%)<br>5 (50%)	0.8964
**Ectropion**	No<br>Yes	14 (63.6%)<br>3 (37.5%)	8 (36.4%)<br>5 (62.5%)	0.3893
**Sleep disorders**	No<br>Yes	13 (65%)<br>4 (40%)	7 (35%)<br>6 (60%)	0.3619
**Inability to fully close the eyelids**	No<br>Yes	10 (58.8%)<br>7 (53.8%)	7 (41.2%)<br>6 (46.2%)	0.9210
**Feeling of ear plugging**	No<br>Yes	10 (58.8%)<br>7 (53.8%)	7 (41.2%)<br>6 (46.2%)	0.9210
**Hearing loss**	No<br>Yes	15 (62.5%)<br>2 (33.3%)	9 (37.5%)<br>4 (66.7%)	0.4071
**Performance status (Karnofsky scale)**	Median [IQR] (Min-Max)	90 [77.5-100]	85 [50-100]	0.5374

* - statistically significant result

Supplementary Table S5. The relationship between personality inventory assessed using the NEO-FFI questionnaire and the occurrence of depression assessed using Beck`s scale in the study group.

Personality Inventory	Level	Depression		*p*
		No [n=17]	Yes [n=13]	
**Openness to experience**	Low<br>Moderate<br>High	1 (50%)<br>4 (50%)<br>12 (60%)	1 (50%)<br>4 (50%)<br>8 (40%)	0.8731
**Neuroticism**	Low<br>Moderate<br>High	1 (100%)<br>10 (90.9%)<br>6 (33.3%)	0 (0%)<br>1 (9.1%)<br>12 (66.7%)	0.0067*
**Agreeableness**	Low<br>Moderate<br>High	0 (0%)<br>2 (25%)<br>15 (71.4%)	1 (100%)<br>6 (75%)<br>6 (28.6%)	0.0400*
**Extroversion**	Moderate<br>High	3 (33.3%)<br>14 (66.7%)	6 (66.7%)<br>7 (33.3%)	0.1983
**Scrupulousness**	Low<br>Moderate<br>High	3 (33.3%)<br>14 (66.7%)	6 (66.7%)<br>7 (33.3%)	0.1983

* - statistically significant result
